# Do Chinese Preschool Children Eat a Sufficiently Diverse Diet? A Cross-Sectional Study in China

**DOI:** 10.3390/nu10060794

**Published:** 2018-06-20

**Authors:** Hua Jiang, Ai Zhao, Wenzhi Zhao, Shengjie Tan, Jian Zhang, Yumei Zhang, Peiyu Wang

**Affiliations:** 1 Department of Nutrition and Food Hygiene, School of Public Health, Peking University Health Science Center, Beijing 100191, China; jianghua@bjmu.edu.cn (H.J.); Owen90806128@163.com (W.Z.); tanshengjie2009@126.com (S.T.); zhangjiantp@163.com (J.Z.); zhangyumei@bjmu.edu.cn (Y.Z.); 2 School of Nursing, Peking University Health Science Center, Beijing 100191, China; 3 Department of Social Medicine and Health Education, School of Public Health, Peking University Health Science Center, Beijing 100191, China; xiaochaai@163.com

**Keywords:** dietary diversity, preschool, nutrient adequacy

## Abstract

Background: This study aimed to comprehensively evaluate dietary diversity and its associated factors in Chinese preschoolers and explore whether the daily food consumption of children with different dietary diversity-associated characteristics met recommended dietary amounts. Methods: A cross-sectional study covering seven cities and two villages was conducted and included 697 preschool children aged 3–7 years old. Dietary diversity score (DDS) and DDS 10 were calculated based on 24-h dietary recall. The food-intake differences among children with different DDS 10 predictors were examined. Results: The mean DDS and DDS 10 in Chinese preschool children were 7.4 ± 1.5 (ranged from 3 to 9) and 7.0 ± 3 (ranged from 3 to 9) respectively. Positive predictors of dietary diversity included residing in an urban environment, a higher household expenditure on children’s food, and a higher frequency of eating outside. Food-intake differences existed among the predictors. Conclusions: Education and intervention should be strengthened to improve the dietary diversity of preschool children, especially in rural areas. The overall dietary pattern of children requires attention, which means not only increasing dietary diversity but also avoiding an unbalanced diet.

## 1. Introduction

The preschool period is crucial for a child’s growth and development, therefore parents pay attention to their children’s nutritional status. It is noteworthy that preschoolers maintain their dietary habits for long periods of time, even into adulthood [1]. Therefore it is important to help children develop and maintain healthy food habits. Many studies have shown that dietary diversification has beneficial effects on micronutrient adequacy and is positively correlated with health outcomes at different ages [2,3,4], especially in children [5,6,7]. For example, some studies have shown significant correlations between dietary diversity and anthropometric measurements (e.g., HAZ (height-for-age *z*-score) and WAZ (weight-for-age *z*-score)) in children [8,9]. In China, similar research has shown a correlation between dietary diversification and growth in children [10,11]. Less diversification in the diet may increase the risk of asthma and allergies in childhood [12]. Randomized controlled trials have also proved that supplementation with multivitamins and iodine may increase nutrient-deficient school-aged children’s intelligence quotient [13]. This evidence indicates that dietary diversification may increase the micronutrient intake and improve children’s physical health and cognitive development. However, recent studies found that only a minority of children had a diverse dietary pattern, and this was especially evident for those in developing countries. For example, less than half of children in rural Burkina Faso received the recommended minimum of four different food groups per day before they were two years old [14], and only 13% of Mexican children and adolescents (5–15 years old) had a diverse dietary pattern [15]. The factors that may influence food diversification need to be explored in order to allow nutritionists to employ improvement measures. Most previous studies focused on the associations of age, gender, district, and socioeconomic status with household dietary patterns [16]. In addition to these influencing factors, eating problems (e.g., picky eating), food allergies, parental self-perception of their child’s nutrition and body image, and out-of-home eating habits should also be taken into consideration. This study aimed to comprehensively measure the dietary diversification and its associated factors in Chinese preschoolers and explore whether the food consumption of children with different dietary diversity-associated characteristics met the recommendation of the Chinese Dietary Guidelines (2016) by Chinese Nutrition Society [17]. The updated guidelines (2016) provide the recommended daily intake amount of essential eight food groups including: (1) cereals and tubers, (2) vegetables, (3) fruits, (4) meat, (5) aquatic products, (6) eggs, (7) dairy products, and (8) soy products and nuts. It is emphasized that dietary diversity is the basic principle of balanced food pattern.

## 2. Subjects and Methods

### 2.1. Subjects

The study protocol was approved by the Ethical Committee of the Health Science Center, Peking University (No. IRB00001052-11042), and written informed consent was obtained from a child’s legal guardian prior to their participation. The study was conducted between 2011 and 2012. For representativeness, seven cities (with different economic levels and in different geographical locations) and two villages (one located in the plains and one located in the mountains) were selected by purposive sampling. One junior, one middle, and one senior class were subsequently randomly selected from a kindergarten in each of the nine districts. The analysis included 697 healthy children aged 3–7 years, and children with congenital diseases or serious illnesses were excluded from the study.

### 2.2. Dietary Data

Food intake data were collected using the 24-h dietary recall method using two questionnaires. One questionnaire was administered by a trained investigator with the help of kindergartens and aimed to collect information on detailed food consumption in the kindergarten. The other questionnaire aimed to interview the parents to collect information on what the children ate at home and away from home, including their dietary supplements. Other details were described in previously published articles [18,19].

### 2.3. Measurement of Dietary Diversity

Two indicators—dietary diversity score (DDS) and DDS 10—were used to evaluate children’s dietary diversity based on the consumption of 10 food groups in the past 24 h according to the guidelines of the Food and Agriculture Organization of the United Nations, as shown in Table 1 [20]. DDS was calculated based on the sum of the total number of food groups without amount requirement, while DDS 10 was calculated with a minimum intake amount requirement. In DDS 10, the child received one point if they consumed at least 10 g from a single food group, except that the threshold for fats and oils was 2 g. Other food groups were not included in the calculation of DDS 10. By adding together the scores of different food groups, the DDS 10 score ranged from 1 to 9.

### 2.4. Sociodemographic and Health-Related Data

Sociodemographic information was collected by interviewing the parents, and included the child’s age, gender, nationality, parental education level, household income and expenditure on the child’s food. Picky eating behavior in this study was defined that the children consumed an inadequate variety and amount of food through rejection of foods that are familiar (and unfamiliar) to them because of specific tastes, textures, smells or appearances. The definition was provided to parents in advance and then they were asked a single question as “Do you consider your child as having picky eating behavior at present?” The frequencies of outside eating, food allergies of children and parents’ perception of their children’s nutrition status and body image were acquired by parent self-report.

### 2.5. Statistical Analysis

The database was established with Epi Data version 3.0 (EpiData Association, Odense, Denmark) and analysis was conducted with IBM SPSS (predictive analytics software and solutions) version 20.0 (International Business Machines Corporation, New York, State of New York, USA). Statistical significance was determined at *p* < 0.05 (two-tailed tests). Descriptive analysis was performed for continuous variables, and results are shown as mean ± standard deviation (SD). Students’ t-tests were used for normally distributed data. For abnormally distributed data, median and quartiles were used to describe the data, and Mann–Whitney U tests were performed to compare the differences. The predictors of DDS 10 were explored by linear regression.

## 3. Results

### 3.1. DDS, DDS 10, and Its Associated Factors

A total of 697 children (97.1% Han Chinese) with a mean age of 4.9 ± 0.9 years (range: 3–7 years) participated in this study. The mean DDS and DDS 10 were 7.4 ± 1.5 (range: 3–9) and 7.0 ± 1.3 (range: 3–9), respectively. For the DDS, a total of 6.2%, 43.6%, and 50.2% of children scored ≤4, 5–7, and >7, respectively. Meanwhile for DDS 10, a total of 6.5%, 60.0%, and 33.6% of children scored ≤4, 5–7, and >7, respectively. The DDS and DDS 10 in children with different sociodemographic characteristics are shown in Table 2. Children aged 6–6.9 years had a significantly lower DDS. Children living in rural areas, whose parents had a lower education level, whose households had a lower income, and whose parents spent less of the household monthly expenditure on their food had significantly lower DDS and DDS 10.

By considering dietary behaviors, food allergy, and the parent’s perception of their child’s nutrition and body image, it was found that children who ate outside more than once per week and whose parents thought they were thin had a significant higher DDS and DDS 10. Meanwhile, children with self-reported uncertain food allergies had a significantly lower DDS 10 (Table 3). Self-reported picky-eating behavior and a parent’s perception of their child’s nutrition were not correlated with DDS or DDS 10.

According to the single factor analysis, the DDS and DDS 10 shared similarly associated factors. Therefore linear regression analysis was only conducted for DDS 10 (Table 4). Finally, living in rural areas was negatively associated with DDS 10, while a higher monthly household expenditure on children’s food and eating outside more than once per week were positively associated with DDS 10.

### 3.2. Food Intake Among Children with Different DDS 10 Predictors

The differences in the daily intake of 10 food groups among children with different DDS 10 predictors were examined. Children living in rural areas had a significantly higher intake of cereals and vegetables; however, they had a significantly lower intake of meat, aquatic products, dairy products, and nuts. Children living in families who spent less of their expenditure on their child’s food showed a significantly higher intake of cereals and vegetables, while children living in households with higher food expenditure showed a significantly higher intake of meat, aquatic products, dairy products, and nuts. Children who were used to eating outside more than once per week showed a higher intake of meat, aquatic products, dairy products, and nuts but a lower intake of cereals and vegetables.

## 4. Discussion

The importance of dietary diversity has been highlighted by researchers and in guidelines [8,17,21,22]. Developing a healthy and balanced dietary pattern is essential for preschool children because their habits in this period may last for a long time [23]. DDS based on food groups are regarded as good indicators of nutrient adequacy and balance [8,20,24], and are more feasible and practical than indicators based on individual foods (e.g., the food variety score). The results from this study showed that DDS 10 had similar correlated factors to DDS. The mean DDS 10 was 7.0 ± 1.30, and 93.6% of children had a DDS 10 ≥5. Similar results were found in other studies; for example, the prevalence of inadequate dietary diversity in children aged 6–59 months was 9% in Sri Lanka [5], and the majority of Ghanaian children (60%) aged 3–6 years consumed a minimum of four out of the seven food groups [9]. The China Health and Nutrition Survey stated that China’s food-consumption patterns have changed dramatically in recent decades [25], therefore people, including children, have more access to different types of foods. In European countries, the mean total Dietary Quality Index (DQI) score was 68.3%, and the mean score of subcomponent was 61.7% for diversity in preschoolers [26]. This shows that the nutritional gap between children in developing and developed countries has declined in recent years.

In this study, living in a rural area was negatively associated with DDS 10, and this result was similar to the findings of other researchers [3,9,10,27]. One important reason for this might be the limited access to variety in rural areas (e.g., due to high electricity and transportation costs, laggard infrastructure development, etc.), which limits dietary diversity [28]. However, the urban–rural disparities in children’s nutritional status in China have mitigated over time due to urbanization [29]. The results from this study also showed that household monthly expenditure on children’s food was positively correlated with DDS, as those parents who pay more attention to their child’s nutrition are more willing to spend more on their food. There were also some associated factors highlighted in the single factor analysis but not in the linear regression analysis (i.e., family income [27,30] and parental education level [26,27,30]), which may be the key factors in a household’s socioeconomic status. No significant associations were found between DDS and gender or age in this study, which meant that both Chinese boys and girls had similar dietary diversification regardless of their age. These findings were different from other research findings; however, the gender difference in DDS in India may have been dependent on gender inequality in the country, which is less significant in China [31]. The results from this study also showed that the frequency of eating outside was positively associated with food diversity. The vast global lifestyle changes since last century mean that out-of-home eating has become very popular for common families, which may, in turn, bring more opportunities to diversify diet [32]. Picky eating, which is a common eating behavior in Chinese children [18], showed no significant association with DDS in this study. We infer that children with a picky-eating problem may refuse single foods (e.g., carrots, mushrooms, etc.) but accept other food types containing similar nutrients, which would therefore not change the results of counting food groups. However, it is still noteworthy that picky eating has negative effects on a child’s growth and development [18,33]. A correlation between food allergies and DDS was also not observed in this current analysis. Parents may consciously offer other food items to supplement what their children are allergic to. In the preschool period, children start to contact the outside environment in kindergarten and community more frequently than toddlers. However, parents still play a leading role in nutrition for preschool children [34]. Interestingly, this study showed that children whose parents thought they were thin had a higher DDS, thus these parents may offer more diverse food types to enable their children to grow stronger.

In addition to analyzing the associated factors of DDS, this study also explored the difference in daily food intake in preschool children. In general, comparing with Chinese dietary guidelines (2016), the invested children had a sufficient food intake of eggs, meat, and soybean products; however, a lower intake of vegetables, fruits, aquatic products, and dairy products was observed. This might indicate unbalanced diets in our study population.

Considering the predictors associated with dietary intake, compared with urban children, rural children ate more cereals and vegetables but ate less meat, aquatic products, dairy products, and nuts. This led to a low DDS, which may consequently result in an insufficient intake of protein, micronutrients (e.g., vitamins, calcium, iodine, phospholipids, etc.), and essential fatty acids. Especially the dairy products intake—which was far lower than the recommendation (2016)—might cause health problems since 60% of the recommended daily allowance (RDA) of calcium comes from dairy calcium. The rural preschoolers with insufficient dairy intake therefore face a higher chance of lower bone-mineral content (BMC) and z-score for bone-mineral density (BMD), as other researchers have demonstrated in their studies [35,36].

Satisfyingly, children with increased levels of food expenditure showed adequate ingestion of aquatic products, dairy products, and nuts; however, they also showed unbalanced diet patterns with insufficient vegetables but excess meat consumption. Food expenditure always reflects the household’s socioeconomic status and the parent’s concern about their child’s nutrition. The higher the parent’s education and income level, the more attention they pay to their child’s nutrition, the greater the household food expenditure. Those well-educated parents tend to offer children meat, aquatic products, dairy products, and nuts which are rich in high protein and micronutrients that are beneficial for physical growth and intelligent development. However, based on the current results, vegetables are to be ignored by those parents. Therefore, proper health advice should urgently be offered, a strategy proven by other researchers to be effective [37,38].

This study also identified contradictory results with regard to outside eating. In this study, outside eating correlated positively with dietary diversity; however, according to the food consumption data (showed in Table 5), it could be inferred that the increasing DDS due to a higher frequency of eating outside had the potential to be caused by the excess intake of meat and aquatic products. Moreover, the preschoolers who ate outside more than once per week consumed far fewer vegetables, and those who ate outside less frequently consumed far fewer dairy products. In addition, the overuse of oil in restaurants and the increased consumption of western foods away from home mean that the hazards of eating out cannot be neglected by parents.

To the best of our knowledge, this multi-center survey is the first study to explore the predictors of dietary diversity in Chinese preschoolers. By analyzing the consumption of 10 food groups, the specific food groups which were associated with the personal characteristics of high risk of lower dietary diversity were identified. It could be of benefit to parents and kindergartens to provide more balanced diets with high dietary diversity and thus contribute to early childhood behavior formation.

There were several limitations to this study. Firstly, the study design was a cross-sectional investigation and it does not, therefore, infer a causal relationship. Secondly, the 24-h dietary record may have caused a recall bias, and one-day recall may lead to missing foods which are not consumed on the reporting day. This limitation could potentially be remedied by the use of repeated 24-h recalls, for example on three days including weekdays and weekends.

The current study explored dietary diversity and its associated factors in Chinese preschoolers, and explored how these factors influenced food intake in children, which is useful for dietary guidance. The suggestions based on the study findings are: (1) education and intervention should be strengthened to improve preschool children’s dietary diversity, especially in rural areas; (2) the overall pattern in children is worthy of attention, which means not only increasing dietary diversity but also avoiding unbalanced diets; and (3) prospective studies are needed to explore the short-term and long-term effects of dietary diversification on growth and development in children.

## Figures and Tables

**Table 1:** Ten food groups of dietary diversity scores.

Food Groups	Foods
1. Cereals, roots and tubers	rice, wheat, maize, cassava, potatoes
2. Vitamin A rich fruits and vegetables	acacia leaves, amaranth, sweet red pepper, Chinese cabbage, carrot, cashew leaf, cassava leaf, chrysanthemum leaf, cowpea leaf, fennel, mustard greens, mango, papaya, etc.
3. Other fruits	apple, banana, orange, pear
4. Other vegetables	cabbage, green beans, broccoli, mushrooms, cucumber, eggplant, cauliflower
5. Legumes, pulses and nuts	beans, peas, peanuts, soy bean
6. Oils and Fats	vegetable oil, lard, butter, ghee
7. Meat, poultry, fish	beef, pork, chicken, turkey, fish, shellfish
8. Dairy	milk, yogurt, cheese, ice cream
9. Eggs	
10. Other food	sweets, chips, soda, condiments, etc.

**Table 2:** The DDS and DDS 10 among Chinese preschool children with different socio-demographic characteristics (Mean ± standard deviation (SD)).

Socio-Demographic Characteristics		*N*	DDS	*P*	DDS 10	*P*
Gender	Male	353	7.4 ± 1.4	0.374	7.1 ± 1.3	0.200
	Female	341	7.3 ± 1.5		6.9 ± 1.4	
Age (years)	3~3.9	122	7.6 ± 1.4	0.036	6.9 ± 1.1	0.095
	4~4.9	237	7.5 ± 1.3		7.1 ± 1.2	
	5~5.9	228	7.2 ± 1.5		7.0 ± 1.4	
	6~6.9	105	7.2 ± 1.7		6.8 ± 1.5	
Living areas	Urban areas	488	8.0 ± 1.1	<0.001	7.4 ± 1.0	<0.001
	Rural areas	209	6.0 ± 1.4		6.0 ± 1.4	
Maternal education	Junior high school or below	259	6.6 ± 1.3	<0.001	6.7 ± 1.4	<0.001
	Senior high school	162	7.0 ± 1.3		7.2 ± 1.4	
	Bachelor’s degree or above	265	7.4 ± 1.1		8.1 ± 1.2	
Paternal education	Junior high school or below	252	6.7 ± 1.4	<0.001	6.5 ± 1.3	<0.001
	Senior high school	149	7.2 ± 1.5		6.9 ± 1.4	
	Bachelor’s degree or above	287	8.2 ± 1.1		7.5 ± 1.0	
Household per capita monthly income (RMB: yuan)	≤2000	232	6.5 ± 1.3	<0.001	6.7 ± 1.5	<0.001
	2001~4000	149	7.3 ± 1.1		7.8 ± 1.3	
	>4000	289	7.5 ± 1.2		8.1 ± 1.5	
Household monthly expenditure on children’s food (RMB: yuan)	≤300	277	6.7 ± 1.5	<0.001	6.5 ± 1.4	<0.001
	301~600	203	7.5 ± 1.3		7.1 ± 1.2	
	>600	150	8.1 ± 1.0		7.5 ± 1.1	

**Table 3:** The DDS and DDS 10 among Chinese preschool children with different dietary behaviors, food allergy and parent’s self-perception of child’s nutrition and body image (Mean ± SD).

Variables		*N*	DDS	*P*	DDS 10	*P*
Picky eating	Yes	370	7.4 ± 1.5	0.165	6.9 ± 1.4	0.343
	No	327	7.5 ± 1.4		7.0 ± 1.2	
Outside eating (times/week)	≤1	194	7.3 ± 1.5	0.020	6.9 ± 1.3	<0.001
	>1	503	7.6 ± 1.3		7.2 ± 1.2	
Food allergy	Yes	62	7.7 ± 1.2	0.052	7.1 ± 1.1	0.020
	No	521	7.4 ± 1.4		7.0 ± 1.3	
	Unsure	114	7.1 ± 1.6		6.7 ± 1.5	
Parent’s self-perception of child’s nutrition	With concern of unbalanced diet	299	7.4 ± 1.5	0.596	7.0 ± 1.4	0.520
	No concern of unbalanced diet	382	7.4 ± 1.4		7.0 ± 1.2	
Parent’s self-perception of child’s body image	Normal	391	7.3 ± 1.5	0.022	6.9 ± 1.4	0.025
	Thin	64	7.8 ± 1.3		7.4 ± 1.2	
	Overweight/obese	236	7.4 ± 1.5		7.0 ± 1.2	

**Table 4:** Linear regression model on predictors of DDS 10 among Chinese preschool children (*N* = 697).

Variables ^a^	*B*	95% CI	*P*
Living in rural areas	−0.98	(−1.26, −0.70)	<0.001
Maternal education level	0.14	(−0.03, 0.31)	0.096
Paternal education level	−0.04	(−0.14, 0.06)	0.419
Household capita monthly income	0.14	(−0.01, 0.29)	0.055
Household monthly expenditure on child’s food	0.15	(0.01, 0.29)	0.039
Food allergy	−0.013	(−0.22, 0.19)	0.896
Outside eating	0.28	(0.07, 0.49)	0.008
Parent’s self-perception of child’s body image	0.02	(−0.08, 0.12)	0.699

^a^ The maternal education level, paternal education level, household capita monthly income and household monthly expenditure on children’s food were categorized as three groups, and assigned values 1, 2 and 3 from the lowest level to the top level respectively; food allergy categorized for three groups as with allergy, without allergy and unsure, and assigned values 1, 2 and 3 respectively; outside eating categorized for two groups, ≤once per week assigned value 1 and >once per week assigned value 2; Parent’s self-perception of child’s body image categorized for three groups as normal, thin and overweight/obese, and assigned values as 1, 2 and 3 respectively.

**Table 5:** Food daily intake among Chinese pre-school children with different characteristics (g/day).

Food Groups	Recommended Amount	Residence		Household Monthly Expenditure on Children’s Food (RMB: yuan)			Outside Eating	
		Urban	Rural	≤300	301~600	>600	≤1 times/week	>1 times/week
		*N* = 498	*N* = 209	*N* = 277	*N* = 203	*N* = 150	*N* = 194	*N* = 503
Cereals	250~400	160.0 (120.0, 224.8)	230.0 (175.0, 340.0) **	200.0 (152.5, 287.5) **	167.5 (125.0, 250.0)	150.0 (120.0, 220.0)	197.5 (150.0, 275.0) *	170.0 (130.0, 250.0)
Tubers		54.3 (37.0, 103.3)	147.1 (0.0, 159.3)	60.0 (0.0, 157.1)	52.9 (16.3, 128.6)	56.9 (40.7, 96.6)	75.8 (0.0, 157.1)	54.3 (27.7, 121.4)
Vegetables	300~500	125.0 (88.0, 209.3)	275.0 (212.5, 450.0) **	212.5 (141.3, 350.0) **	160.0 (100.0, 260.7)	129.2 (95.0, 226.1)	218.9 (138.8, 325.6) **	160.0 (100.0, 260.0)
Fruits	200~350	150.0 (109.5, 250.0)	184.3 (30.0, 250.0)	161.4 (50.0, 250.0)	160.0 (109.4, 250.0)	157.9 (110.0, 223.4)	164.3 (57.1, 250.0)	150.0 (85.7, 250.0)
Meat	40~75	80.0 (50.0, 130.0)	34.3 (26.7, 51.4) **	45.0 (27.1, 78.6)	65.0 (37.5, 110.00	88.6 (49.5, 140.0) **	48.6 (27.8, 91.4)	65.0 (37.5, 114.3) **
Aquatic products	40~75	56.9 (6.8, 107.9)	0.0 (0.0, 0.0) **	0.0 (0.0, 22.7)	26.0 (0.0, 103.6)	51.5 (3.0, 100.0) **	0.0 (0.0, 56.6)	26.9 (0.0, 98.6) **
Eggs	40~50	72.1 (431.0, 105.0)	70.0 (0.0, 110.0)	70.0 (140.0, 100.0)	72.1 (35.7, 110.0)	68.9 (38.8, 110.0)	75.8 (41., 120.0)	70.0 (34.3, 105.0)
Dairy products	300	241.0 (74.9, 370.0)	7.5 (0.0, 85.7) **	42.9 (0.0, 225.0)	200.0 (25.0, 314.3)	250.0 (110.0, 400.0) **	83.6 (0.0, 250.0)	200.0 (8.6, 325.0) **
Soy products	25~35	31.1 (19.1, 64.3)	33.3 (0.0, 78.6)	26.7 (0.0, 68.5)	31.4 (13.6, 62.9)	34.3 (21.7, 68.0)	35.3 (0.0, 70.0)	29.6 (15.5, 64.3)
Nuts		8.6 (25.5, 25.0)	0.0 (0.0, 4.6) **	1.3 (0.0, 9.6)	5.0 (0.07, 21.3)	7.1 (2.6, 21.5) *	3.3 (0.0, 9.2)	5.7 (0.3, 22.0) **

** The highest intake group *p* < 0.001; * the highest intake group *p* < 0.05.

## References

[1] Kaikkonen J.E., Mikkilä V., Magnussen C.G., Juonala M., Viikari J.S., Raitakari O.T. (2013). Does Childhood Nutrition Influence Adult Cardiovascular Disease Risk? Insights from the Young Finns Study. Ann. Med..

[2] Isa F., Xie L.P., Hu Z.Q., Zhong Z.H., Hemelt M., Reulen R.C., Wong Y.C., Tam P.C., Yang K., Chai C. (2013). Dietary Consumption and Diet Diversity and Risk of Developing Bladder Cancer: Results from the South and East China Case-Control Study. Cancer Causes Control.

[3] Jin Y. (2009). Study on Associations of Dietary Diversity with Nutrients Adequacy and Nutrition Related Chronic Disease in Chinese Adults.

[4] Otsuka R., Kato Y., Nishita Y., Tange C., Nakamoto M., Tomida M., Imai T., Ando F., Shimokata H., Suzuki T. (2016). Dietary Diversity and 14-Year Decline in Higher-Level Functional Capacity Among Middle-Aged and Elderly Japanese. Nutrition.

[5] Perkins J.M., Jayatissa R., Subramanian S.V. (2018). Dietary Diversity and Anthropometric Status and Failure among Infants and Young Children in Sri Lanka. Nutrition.

[6] Fernandez C., Kasper N.M., Miller A.L., Lumeng J.C., Peterson K.E. (2016). Association of Dietary Variety and Diversity with Body Mass Index in US Preschool Children. Pediatrics.

[7] Rocha N.P., Milagres L.C., Longo G.Z., Ribeiro A.Q., Novaes J.F. (2017). Association Between Dietary Pattern and Cardiometabolic Risk in Children and Adolescents: A Systematic Review. J. Pediatr..

[8] Frempong R.B., Annim S.K. (2017). Dietary Diversity and Child Malnutrition in Ghana. Heliyon.

[9] Steyn N.P., Nel J.H., Nantel G., Kennedy G., Labadarios D. (2006). Food Variety and Dietary Diversity Scores in Children: Are They Good Indicators of Dietary Adequacy?. Public Health Nutr..

[10] Zhang Q., Wan Q.Q., Yu S.Y., Liu Z.T., Zhao J., Li J.J., Wang X.W., Ruan Y., Wan R. (2015). Correlation of Diet Diversity and Growth of Children Aged 2–6 Years Old in Poor Areas, Yunan. Chin. J. Child Health Care.

[11] Zhang J.X., Shi L., Wang J., Wang Y. (2009). An Infant and Child Feeding Index is Associated with Child Nutritional Status in Rural China. Early Hum. Dev..

[12] Nwaru B.I., Takkinen H., Kaila M., Erkkola M., Ahonen S.A., Pekkanen J., Simell O., Veijola R., Ilonen J., Hyöty H. (2014). Food Diversity in Infancy and the Risk of Childhood Asthma and Allergies. J. Allergy Clin. Immun..

[13] Protzko J., Raising I.Q. (2017). Among School-Aged Children: Five Meta-Analyses and a Review of Randomized Controlled Trials. Dev. Rev..

[14] Sawadogo S.P., Yves M., Claire M., Alain B., Alfred T.S., Serge T., Francis D. (2010). Late Introduction and Poor Diversity were the Main Weaknesses of Complementary Foods in a Cohort Study in Rural Burkina Faso. Nutrition.

[15] Galvan-Portillo M., Sánchez E., Cardenas-Cardenas L.M., Karam R., Claudio L., Cruz M., Burguete-Garcia A.I. (2018). Dietary Patterns in Mexican Children and Adolescents: Characterization and Relation with Socioeconomic and Home Environment Factors. Appetite.

[16] Amugsi D.A., Mittelmark M.B., Oduro A. (2015). Association between Maternal and Child Dietary Diversity: An Analysis of the Ghana Demographic and Health Survey. PLoS ONE.

[17] Chinese Nutrition Society (2016). Chinese Dietary Guidelines.

[18] Xue Y., Zhao A., Cai L., Yang B., Szeto I.M., Ma D., Zhang Y., Wang P. (2015). Growth and Development in Chinese Pre-Schoolers with Picky Eating Behaviour: A Cross-Sectional Study. PLoS ONE.

[19] Zhao W., Yu K., Tan S., Zheng Y., Zhao A., Wang P., Zhang Y. (2017). Dietary Diversity Scores: An Indicator of Micronutrient Inadequacy Instead of Obesity for Chinese Children. BMC Public Health.

[20] Kennedy G., Nantel G. (2006). Basic Guidelines for Validation of a Simple Dietary Diversity Score as an Indicator of Dietary Nutrient Adequacy for Non-Breastfeeding Children 2–6 Years.

[21] Azadbakht L., Esmaillzadeh A. (2012). Dietary Energy Density is Favorably Associated with Dietary Diversity Score among Female University Students in Isfahan. Nutrition.

[22] Huang Y.C., Wahlqvist M.L., Lee M.S. (2014). Appetite Predicts Mortality in Free-Living Older Adults in Association with Dietary Diversity. A NAHSIT Cohort Study. Appetite.

[23] Jarman M., Ogden J., Inskip H., Lawrence W., Baird J., Cooper C., Robinson S., Barker M. (2015). How do Mothers Manage their Preschool Children’s Eating Habits and Does this Change as Children Grow Older? A Longitudinal Analysis. Appetite.

[24] Gewa C.A., Murphy S.P., Weiss R.E., Neumann C.G. (2014). Determining Minimum Food Intake Amounts for Diet Diversity Scores to Maximize Associations with Nutrient Adequacy: An Analysis of Schoolchildren’s Diets in Rural Kenya. Public Health Nutr..

[25] Zhai F.Y., Du S.F., Wang Z.H., Zhang J.G., Du W.W., Popkin B.M. (2014). Dynamics of the Chinese Diet and the Role of Urbanicity, 1991–2011. Obes. Rev..

[26] Pinket A., De Craemer M., Huybrechts I., De Bourdeaudhuij I., Deforche B., Cardon G., Androutsos O., Koletzko B., Moreno L., Socha P. (2016). Diet Quality in European Pre-Schoolers: Evaluation Based on Diet Quality Indices and Association with Gender, Socio-Economic Status and Overweight, the ToyBox-Study. Public Health Nutr..

[27] Gausman J., Perkins J.M., Lee H.Y., Mejia-Guevara I., Nam Y.S., Lee J.K., Oh J., Subramanian S.V. (2018). Ecological and Social Patterns of Child Dietary Diversity in India: A Population-Based Study. Nutrition.

[28] Liu J., Shively G.E., Binkley J.K. (2014). Access to Variety Contributes to Dietary Diversity in China. Food Policy.

[29] Liu H., Fang H., Zhao Z. (2013). Urban-Rural Disparities of Child Health and Nutritional Status in China from 1989 to 2006. Econ. Hum. Biol..

[30] Hirvonen K. (2016). Rural–Urban Differences in Children’s Dietary Diversity in Ethiopia: A Poisson Decomposition Analysis. Econ. Lett..

[31] Aurino E. (2017). Do Boys Eat Better than Girls in India? Longitudinal Evidence on Dietary Diversity and Food Consumption Disparities among Children and Adolescents. Econ. Hum. Biol..

[32] Adams J., Goffe L., Brown T., Lake A.A., Summerbell C., White M., Wrieden W., Adamson A.J. (2015). Frequency and Socio-Demographic Correlates of Eating Meals Out and Take-Away Meals at Home: Cross-Sectional Analysis of the UK National Diet and Nutrition Survey, Waves 1-4 (2008-12). Int. J. Behav. Nutr. Phys. Act..

[33] Dubois L., Farmer A.P., Girard M., Peterson K. (2007). Preschool Children’s Eating Behaviours are Related to Dietary Adequacy and Body Weight. Eur. J. Clin. Nutr..

[34] Metcalfe J.J., Fiese B.H. (2018). Family Food Involvement is Related to Healthier Dietary Intake in Preschool-Aged Children. Appetite.

[35] Huncharek M., Muscat J., Kupelnick B. (2008). Impact of dairy products and dietary calcium on bone-mineral content in children: Results of a meta-analysis. Bone.

[36] Infante D., Tormo R. (2000). Risk of inadequate bone mineralization in diseases involving long-term suppression of dairy products. J. Pediatr. Gastroenterol. Nutr..

[37] Darrouzet-Nardi A.F., Miller L.C., Joshi N., Mahato S., Lohani M., Rogers B.L. (2016). Child Dietary Quality in Rural Nepal: Effectiveness of a Community-Level Development Intervention. Food Policy..

[38] Quandt S.A., Grzywacz J.G., Trejo G., Arcury T.A. (2014). Nutritional Strategies of Latino Farmworker Families with Preschool Children: Identifying Leverage Points for Obesity Prevention. Soc. Sci. Med..

